# Dr. Frank Leon Waldorf

**Published:** 1917-10

**Authors:** 


**Dr. Frank Leon Waldorf,** Syracuse 1910, of East Syracuse, died at the Hospital of the Good Shepard, Syracuse, Aug. 14, aged 30, of septicaemia due to an infection of the finger at a necropsy 4 weeks previously. He was Pathologist to the Hospital.

